# Supplementary material for: Resilience Protects Nurses from Workplace Gaslighting and Quiet Quitting, and Improves Their Work Engagement: A Cross-Sectional Study in Greece
Source: Healthcare (Basel). 2025 Aug 20;13(16):2064. doi: 10.3390/healthcare13162064 (PMC12386001; doi:10.3390/healthcare13162064)
Supplement: Supplementary file 1 [file healthcare-13-02064-s001.zip › healthcare-3813018-supplementary.pdf]

Supplementary material

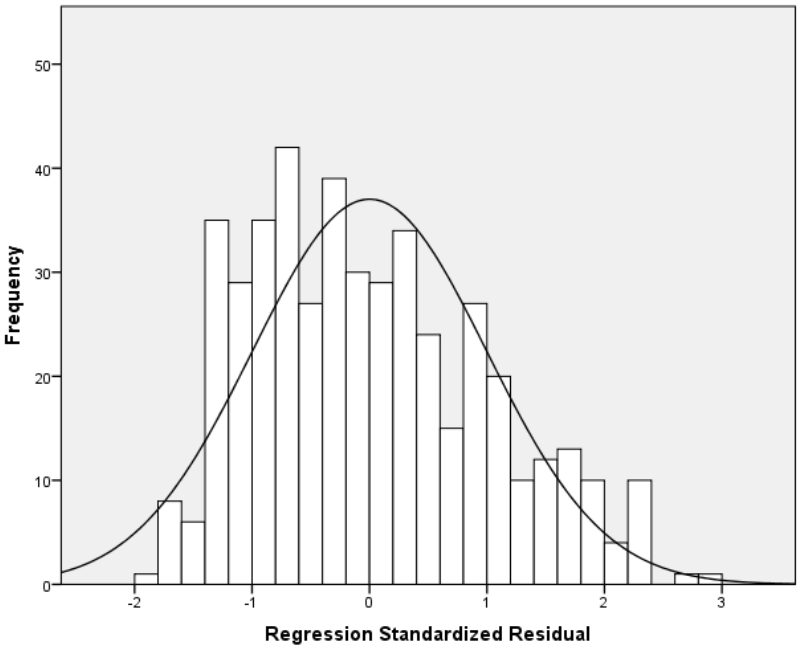

Figure S1. Histogram of the residuals with loss of self-trust as the dependent variable.

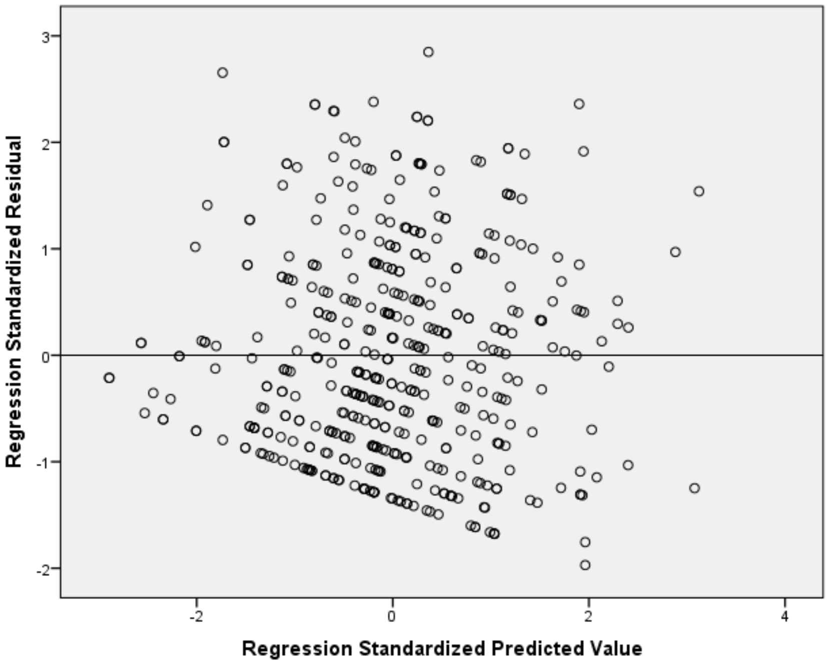

Figure S2. Scatterplot of residuals versus predicted values with loss of self-trust as the dependent variable.

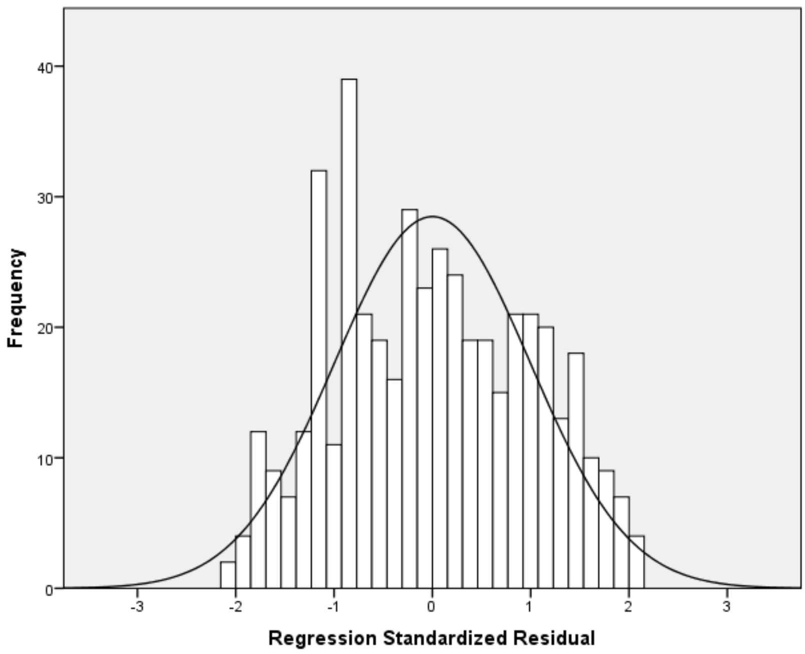

Figure S3. Histogram of the residuals with abuse of power as the dependent variable.

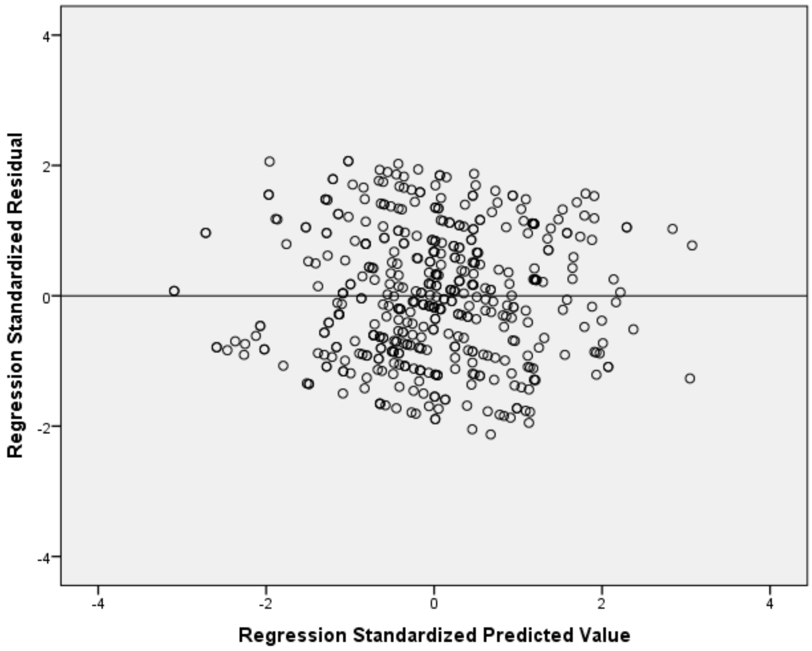

Figure S4. Scatterplot of residuals versus predicted values with abuse of power as the dependent variable.

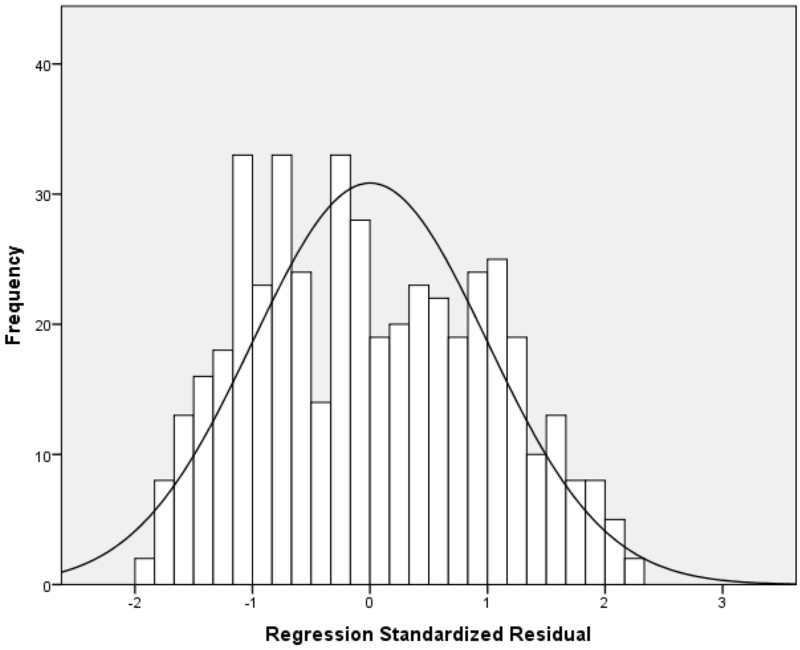

Figure S5. Histogram of the residuals with workplace gaslighting score as the dependent variable.

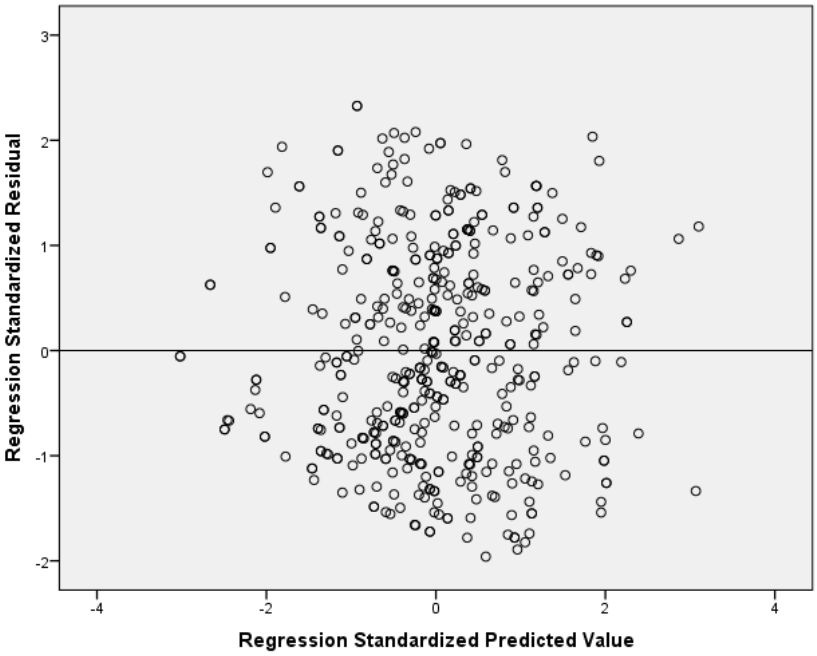

Figure S6. Scatterplot of residuals versus predicted values with workplace gaslighting score as the dependent variable.

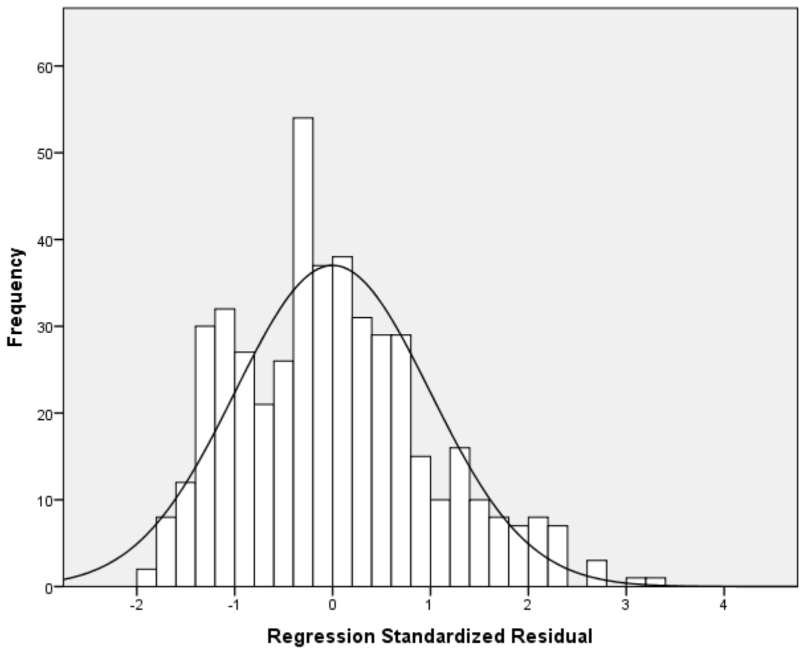

Figure S7. Histogram of the residuals with detachment as the dependent variable.

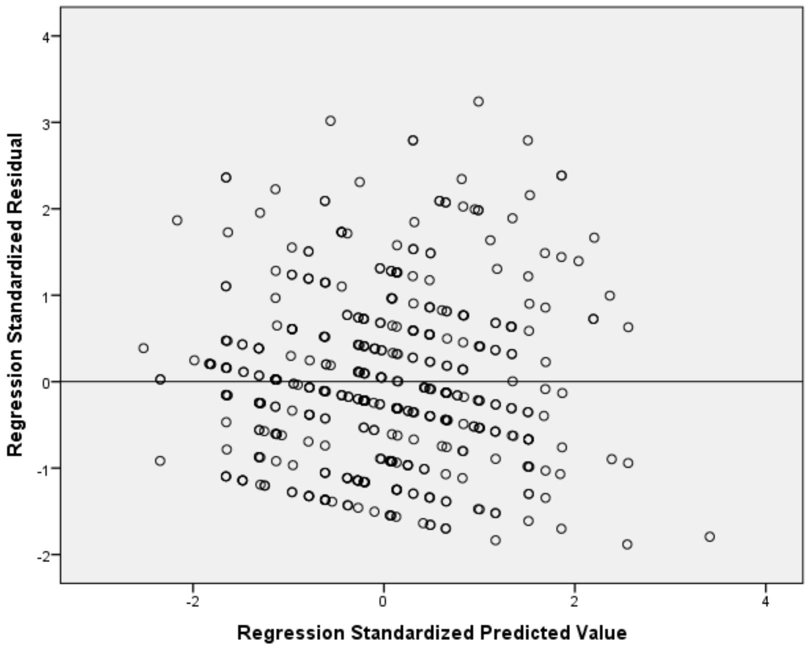

Figure S8. Scatterplot of residuals versus predicted values with detachment as the dependent variable.

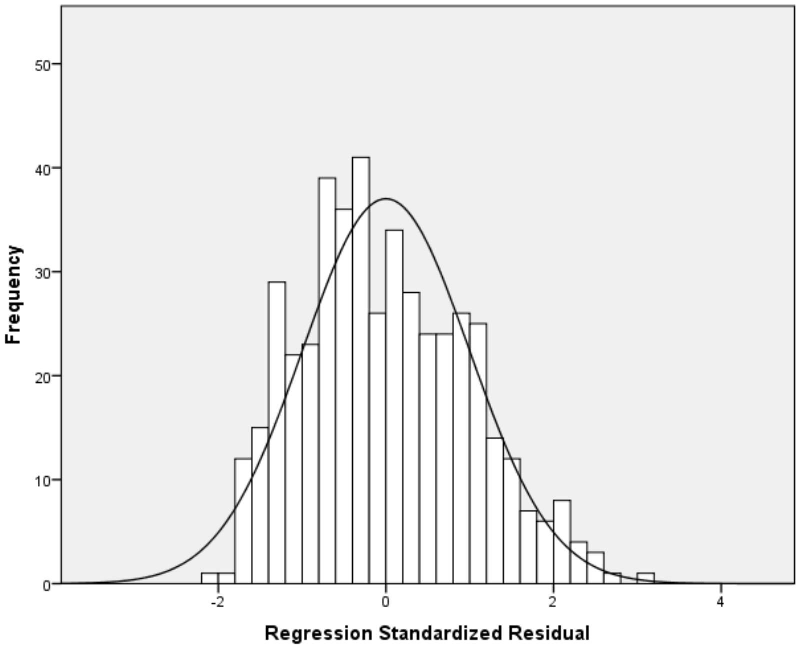

Figure S9. Histogram of the residuals with lack of initiative as the dependent variable.

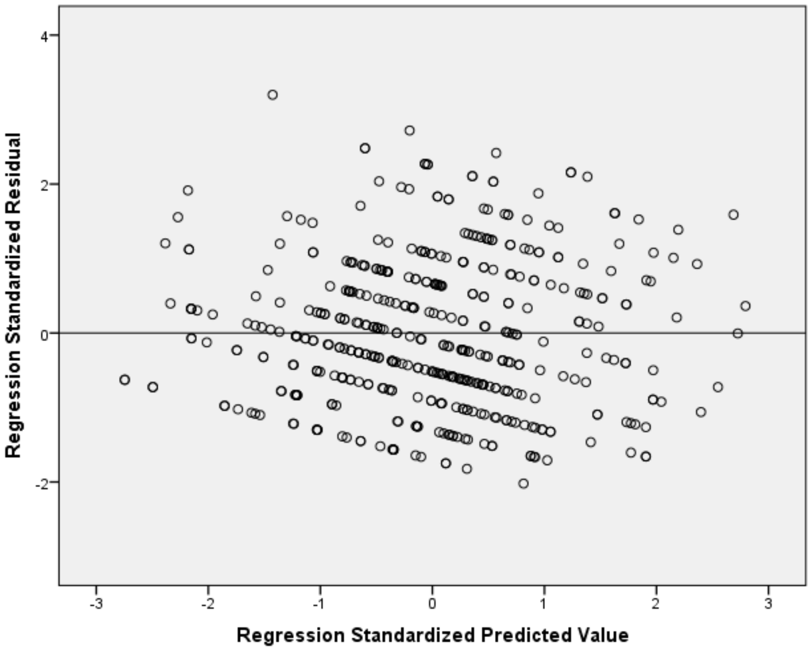

Figure S10. Scatterplot of residuals versus predicted values with lack of initiative as the dependent variable.

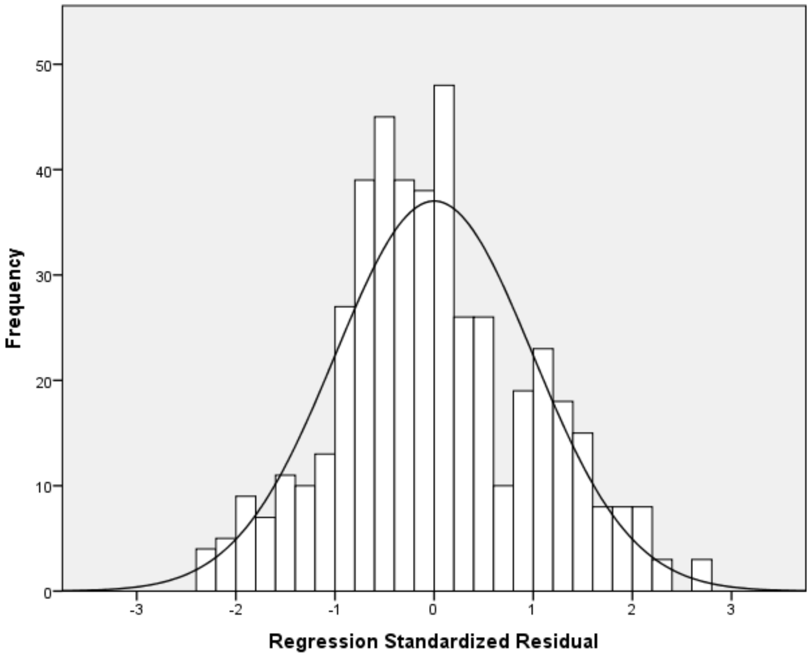

Figure S11. Histogram of the residuals with lack of motivation as the dependent variable.

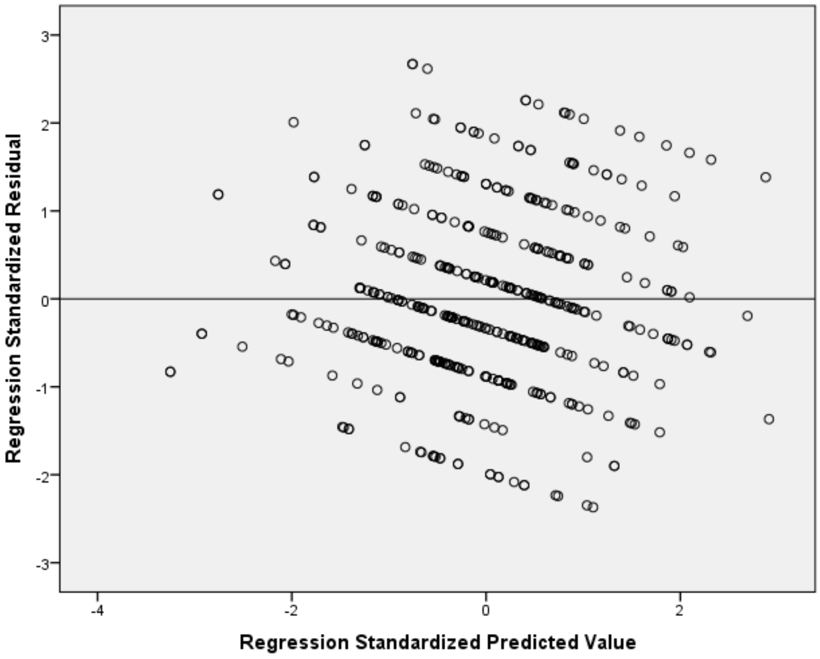

Figure S12. Scatterplot of residuals versus predicted values with lack of motivation as the dependent variable.

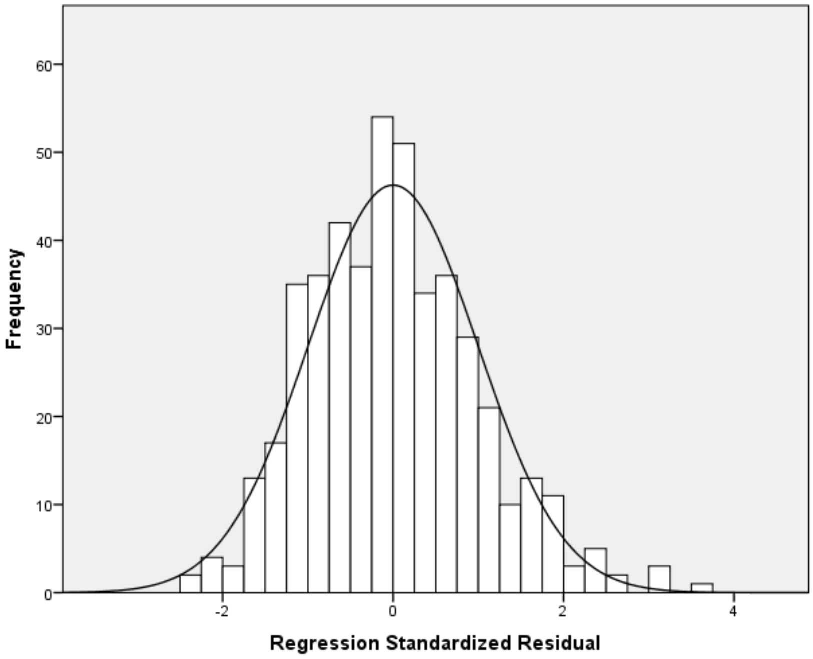

Figure S13. Histogram of the residuals with quiet quitting score as the dependent variable.

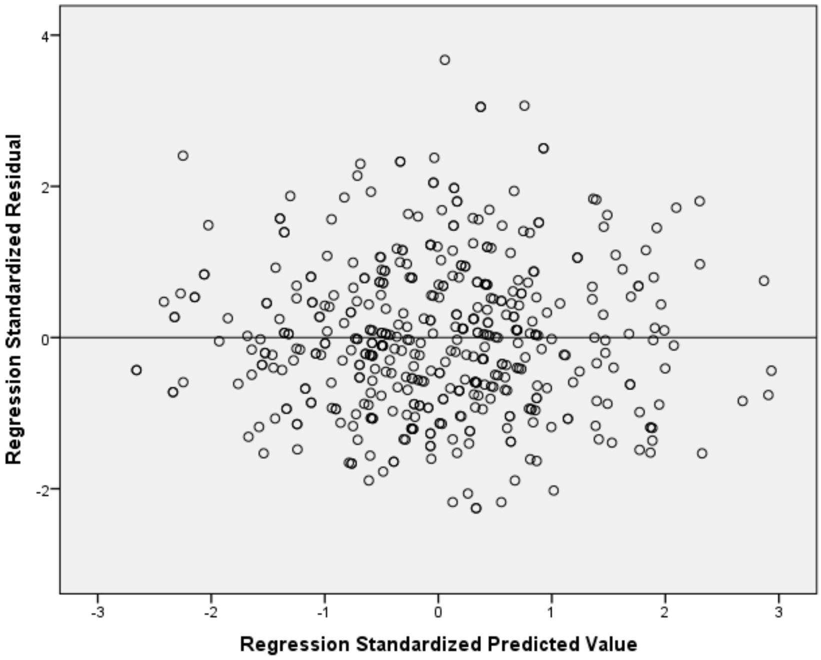

Figure S14. Scatterplot of residuals versus predicted values with quiet quitting score as the dependent variable.

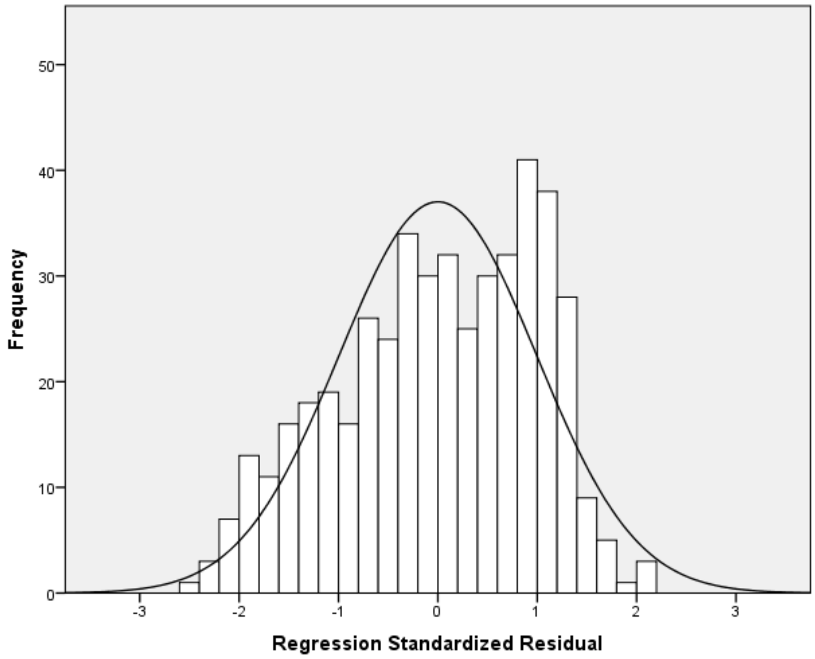

Figure S15. Histogram of the residuals with work engagement score as the dependent variable.

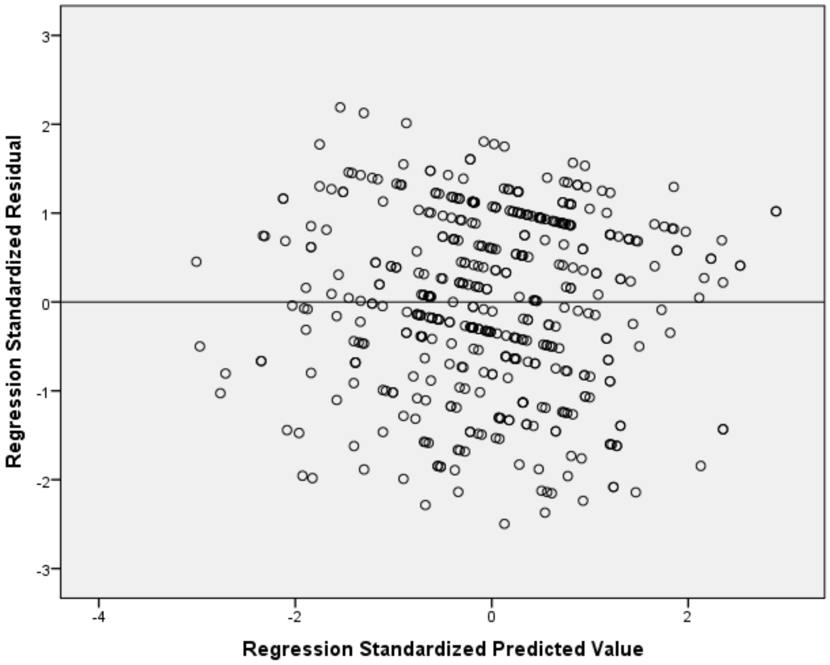

Figure S16. Scatterplot of residuals versus predicted values with work engagement score as the dependent variable.
